# Caregiver burden and the associated factors in the family caregivers of patients with schizophrenia

**DOI:** 10.1002/nop2.1205

**Published:** 2022-03-28

**Authors:** Farnaz Rahmani, Fariborz Roshangar, Leila Gholizadeh, Elnaz Asghari

**Affiliations:** ^1^ Research Center of Psychiatry and Behavioral Sciences Tabriz University of Medical Sciences Tabriz Iran; ^2^ 48432 Department of Medical‐Surgical Nursing School of Nursing and Midwifery Tabriz University of Medical Sciences Tabriz Iran; ^3^ Faculty of Health University of Technology Sydney New South Wales Australia

**Keywords:** burden, caring, family, mental disorder

## Abstract

**Aim:**

Our study aimed to investigate the caregiving burden and its associated factors in family caregivers of patients with schizophrenia.

**Design:**

Correlational study.

**Method:**

Using the convenience sampling method, 215 caregivers were recruited from outpatient clinics affiliated with a tertiary referral psychiatric hospital in Iran. The caregiving burden was measured by the Zarit Burden Interview (ZBI‐22), and associations between caregiving burden and potential factors were examined using multiple regression analysis. We used the STROBE checklist to report the results.

**Results:**

Family caregivers of patients with schizophrenia reported a high level of caregiving burden, with 38.2% of the caregivers perceiving severe burden relating to their role. In the regression analysis, age, gender, educational level, income, job loss due to caregiving, relationship with patient, disease duration and frequency of caregiving were statistically significant predictors of caregiving burden. The regression model explained 54.4% of the variance of caregiving burden.

## 1 INTRODUCTION

Schizophrenia is a severe and chronic mental disease affecting approximately 20 million people globally (World Health Organization, 2019). It is a debilitating disease, causing moderate‐to‐severe impairments in the patient's thinking, perceptions and behaviours (Cannon, 2015). Various aspects of the patient's life are disrupted, including interpersonal, social, job, education and self‐care (Jacob, 2015).

A chronic disease in a family member, particularly a severe mental illness, can adversely affect the function of the whole family, particularly in terms of career success and social or recreational activities. Like an uninvited guest, a chronic health condition disrupts the vital balance of the family system in relationships, goals, expectations, aspirations and hopes (Dehghani et al., 2019). Patients with schizophrenia are often dependent on their caregivers due to the statistically significant impairments associated with their disease. Families are expected to provide the majority of support; they spend statistically significant time caring for the mentally ill family member while living in constant fear of relapse and worrying about an impact of the disease on other family members (Patel & Chatterji, 2015).

Prolonged caregiving responsibilities can deplete the family's energy and increase negative emotions, such as feelings of despair, guilt, depression and helplessness in the family members (Leng et al., 2019). They face unpredictable stressors, including the patient's bizarre behaviours. External stressors, such as stigma and discrimination, are also common, leading to self‐isolation and feelings of loneliness (Akbari et al., 2018). These circumstances upsurge the risk of mental disorders in the family members, particularly among parents and spouses who often take on primary caregiving responsibilities (Von Kardorff et al., 2016).

## 2 BACKGROUND

Long‐term caring responsibilities can cause the caregiving burden, affecting caregivers' well‐being, quality of life, career, social activities and personal relationships (Mulud & McCarthy, 2017; Ribé et al., 2018). Families may experience moderate‐to‐severe care‐related stress in adapting to the needs of a patient with severe mental illness (Souza et al., 2017). Long‐lasting stress can diminish the family's capacity to cope effectively with the challenges (Schetter & Dolbier, 2011). The caregiving burden is a multi‐dimensional concept that adversely affects caregivers' health and well‐being, interpersonal relationships and roles and responsibilities (Liu et al., 2020). It increases stress vulnerability and reduces resilience, leading to mental health issues in the caregivers (Shamsaei, Cheraghi & Bashirian, 2020). There is compelling evidence that family caregivers of patients with mental disease suffer from high levels of stress but receive little support (Akbari et al., 2018; Alzahrani et al., 2017). Many family caregivers have unmet care needs that lead to the caregiving burden (Doody et al., 2017; Hamann & Heres, 2019).

The caregiving burden has gained statistically significant research attention over the last decade. Evidence indicates that family caregivers of mentally ill patients, in particular, schizophrenia experience considerable challenges that often lead to the caregiving burden, negatively affecting their health and well‐being and compromising caregiving quality. Research is scarce on factors that contribute to the caregiving burden in schizophrenia. Mental healthcare providers often pay no attention to the needs of caregivers of patients with schizophrenia (Rahmani et al., 2018), leaving them alone to deal with their ongoing encounters and challenges. Knowledge about caregiver burden in schizophrenia is essential to assist healthcare professionals in supporting and empowering families in their caregiving role and reducing the caregiving burden.

## 3 METHODS

### 3.1 Design

We used a correlational study design to investigate the caregiving burden and the associated factors in family caregivers of patients with schizophrenia.

### 3.2 Participants and settings

Using the convenience sampling method, 215 participants were recruited from outpatient clinics affiliated with Razi Psychiatric Hospital between December 2018 and April 2019. Razi Psychiatric Hospital is an 830‐bed teaching tertiary referral hospital in the northwest of Iran. Study inclusion criteria included being: (a) 18 years or older, (b) the primary family caregiver for a patient with schizophrenia and (c) involved in the caregiving experience for at least one year. A primary caregiver was defined as someone who most met the needs of the patient with schizophrenia, including their physical, emotional, financial, caring, social, homemaking and other needs on a daily or intermittent basis. Family caregivers were excluded if they had a history of psychiatric disease based on their self‐report, or the patient, whom they were providing caregiving, had a comorbid disease/s or intellectual disability.

### 3.3 Instrument

We used the Zarit Burden Interview (ZBI‐22) to assess the caregiving burden. The ZBI is the commonly used tool for assessing the caregiving burden in clinical and research settings. It is a free public access scale. The ZBI‐22 consists of 22 items, addressing caregiver burden in the five domains of relationship burden (6 items), emotional and well‐being (7 items), social and family life (4 items), finances (1 item) and loss of control over life (4 items) (Zarit et al., 1985). The ZBI‐22 uses a 5‐point Likert scale with responses ranging from 0 = never, 1 = rarely, 2 = sometimes, 3 = quite frequently and 4 = nearly always. Total scores range from 0 to a maximum score of 88 (Zarit et al., 1985); higher scores imply greater caregiver burden. The recommended cut‐offs points for the ZBI‐22 are as follows: 0–21 (little or no burden), 21–40 (mild to the moderate burden), 41–60 (moderate‐to‐severe burden) and 61–88 (severe burden) (Zarit et al., 1985). The scale has shown excellent internal consistency across different population groups, with the reported Cronbach's alpha coefficients ranging from 0.77 to 0.93 (Adib‐Hajbaghery & Ahmadi, 2019; Siddiqui & Khalid, 2019). For the Iranian version, Cronbach's alpha coefficient and the test–retest reliability were .94 and *r* = .84 respectively (Navidian et al., 2012).

We collected information on the demographics of both participants and patients, such as age, gender, level of education, employment status, relationship with the patient, perceived income adequacy, duration of disease and duration of caregiving. The survey package was reviewed by an expert panel of 11 faculty members from Tabriz University of Medical Sciences, who confirmed the face and content validity of the survey.

### 3.4 Ethics

The study received approval from the Ethics Committee of Tabriz University of Medical Sciences (TBZMED.REC.1394.968). Potential participants were informed about the purpose of the study, and those interested signed written consent. Participation in the study was voluntary; researchers ensured the privacy and anonymity of the participants. The survey was distributed to eligible participants while awaiting their patients' follow‐up visits with a psychiatrist. All participants completed the survey and returned it to the researcher before leaving the outpatient clinics.

### 3.5 Data analysis

Data were analysed using the Statistical Program for the Social Sciences (SPSS) version 13.0 for Windows. The Kolmogorov‐ Smirnov test was used to examine the data distribution. Differences in the means of the ZBI scores according to demographic and disease variables were assessed using the independent sample *t*‐tests or the one‐way analysis of variance (ANOVA). The contribution of the caregiver‐ and the patient‐related variables to caregiving burden was examined using the multiple regression analysis. The following independent variables were included in the regression analysis: caregiver's age, gender, education, relationship with the patient, employment status, job loss due to caregiving, perceived income adequacy, duration of disease and frequency of caregiving. The sample size of 215 participants was considered adequate to examine the nine independent variables in the regression analysis (Pallant, 2020). Statistical significance for all tests was set at *p* < .05.

## 4 RESULTS

### 4.1 Demographic characteristics

The demographic characteristics of caregivers and patients are presented in Table 1. The mean age of participants was 41.03 ± 10.82 years. More participants were female (57.0%), studied up to high school (32.0%), and the spouse of the patient (52.0%), and perceived their income as inadequate (60.44%). Most of the participants were unemployed (57.0%) and 29.3% lost their job due to caregiving responsibilities. The average disease duration was 13.24 ± 4.66 years, and 57.0% of participants provided caregiving for more than ten years (57.0%), with 48.4% of participants providing caregiving continuously and daily. Patients were mainly male (72.0%), with an average age of 43.24 ± 14.32 years and an average disease duration of 26.46 ± 9.14 years.

**TABLE 1:** Demographics of the study participants and their patients

Characteristics	*N* (%)
Age (years)	
≤45	122 (56.7)
>45	93 (43.3)
Gender	
Male	94 (43.7)
Female	121 (56.3)
Level of education	
No formal education	22 (10.2)
Elementary	65 (30.2)
Secondary	53 (24.7)
High School	70 (32.6)
University	5 (2.3)
Relationship with the patient	
Spouse	113 (52.5)
Parent	52 (24.2)
Child	29 (13.5)
Sibling	14 (6.5)
Frequency of caregiving	
Only weekends	37 (17.2)
Daily but during specific hours	72 (33.5)
Continuously and daily	105 (48.8)
Employment status	
Employed	94 (43.7)
Unemployed	121 (56.3)
If you are unemployed, have you lost your job due to caregiving?	
Yes	63 (29.3)
No	58 (27.0)
Patient's age	
<45	157 (73.0)
>45	58 (26.0)
Patient's gender	
Male	153 (71.2)
Female	62 (28.8)
Duration of disease	
1–10 years	85 (39.5)
>10 years	130 (60.5)

### 4.2 Caregiving burden

The mean of ZBI scores was 65.14 ± 9.17, suggesting a severe caregiving burden. Severe caregiving burden (the ZBI scores 61–88) was reported by 38.2% of participants, 29.4% perceived moderate‐to‐severe caregiving burden (the ZBI scores 41–60), and 19.39% reported mild‐to‐moderate burden (the ZBI scores 21–40). Only 12.9% of the caregivers reported little or no burden (the ZBI scores 0–21) (Table 2).

**TABLE 2:** Perceived caregiving burden in the caregivers

Caregiver burden	*N* (%)
Little or no burden	28 (12.9)
Mild to moderate	41 (19.4)
Moderate to severe	63 (29.4)
Severe burden	82 (38.3)

### 4.3 Perceived caregiving burden according to the caregivers and patients’ characteristics

The mean of ZBI scores was higher in caregivers who were above 40 years of age (58.1 ± 9.7, *p* < .001), female (49.1 ± 9.4, *p* < .001), spouse caregivers (59.2 ± 9.2, *p* < .001) and had no formal education (60.1 ± 9.7, *p* < .01). The mean of ZBI scores was also higher in unemployed caregivers (54.1 ± 9.3, *p* < .001), those who lost their job due to caregiving (57.4 ± 8.4, *p* < .01), perceived their income as inadequate (53.0 ± 8.1, *p* < .001) and provided caregiving continuously and daily (58.2 ± 9.6, *p* < .001). In addition, the mean of ZBI scores was higher in the caregivers of patients with longer disease duration (59.2 ± 8.6, *p* < .01). There was no statistically significant difference in the caregiving burden according to the patient's age or gender (Table 3).

**TABLE 3:** Perceived caregiving burden according to the caregivers and patients’ characteristics

Variables	Mean (SD)	*p* value
Caregiver's age		
<45 years	41.2 ± 8.3	<.001*, *r* = .4
>45 years	58.1 ± 9.7	
Gender		
Male	35.1 ± 8.2	<.001**, *t* = 2.4
Female	49.1 ± 9.4	
Level of education		
No formal education	60.1 ± 9.7	<.01***, *F* = 2.1
Elementary	54.2 ± 9.3	
Secondary	51.2 ± 9.1	
High School	45.1 ± 8.7	
University	38.2 ± 8.2	
Relationship with the patient		
Spouse	59.2 ± 9.2	<.001***, *F* = 3.2
Parent	51.3 ± 8.9	
Child	46.4 ± 8.2	
Sibling	39.2 ± 7.8	
Employment status		
Employed	38.1 ± 7.4	<.001**, *t* = 3.9
Unemployed	54.1 ± 9.3	
Adequate income		
Yes	37.2 ± 6.3	<.001**, *t* = 3.4
No	53.0 ± 8.1	
Frequency of caregiving		
On the weekends	51.3 ± 8.	<.01***, *F* = 3.6
Specific hours daily	56.7 ± 9.1	
Daily & continuously	58.2 ± 9.6	
If you are unemployed, have you lost your job due to caregiving?		
Yes	57.4 ± 8.4	<.01**, *t* = 2.7
No	46.3 ± 7.4	
Patient's age		
<45 years	37.3 ± 10.7	.06*, *r* = −.13
>45 years	36.4 ± 10.9	
Patient's gender		
Male	41.3 ± 8.3	.9*, *t* = 0.17
Female	42.7 ± 8.4	
Duration of disease		
<10 years	42.3 ± 7.2	<.001*, *r* = .5
>10 years	59.2 ± 8.6	

Note

*p is the result of the Pearson correlation, **p is the result of the *t* test and ***p is the result of the ANOVA.

In the regression analysis, caregivers’ age, gender, education, relationship with the patient, job loss due to caregiving, perceived income, frequency of caregiving and disease duration remained statistically significant correlates of the caregiving burden (Table 4). The overall regression model explained 54.4% of the variance of the caregiving burden.

**TABLE 4:** Predictors of caregiver burden in the multiple linear regression analysis

Independent variables	Unstandardized Coefficients (β)	Std. Error	Standardized Coefficients (beta)	*t*	*p* value	*R* ^2^
Constant	−4.390	6.41		−0.651	.522	.544
Caregiver's age	1.193	3.73	−0.144	−2.263	.021	
Female gender	4.181	2.39	0.173	2.471	<.001	
Education	4.673	2.41	0.156	2.214	.039	
Relationship with the patient	6.839	2.36	0.149	2.645	.041	
Employment status	−3.741	4.62	−0.183	−2.527	.798	
Perceived adequate income	−2.320	0.17	0.164	6.545	<.001	
Frequency of caregiving	3.349	2.41	0.193	2.271	<.001	
Duration of disease	−4.736	3.78	−0.167	4.482	<.001	
Job loss	−4.847	2.91	−0.192	3.451	.012	

## 5 DISCUSSION

Participants in this study experienced a high level of burden concerning their caregiving role, with 38.2% of the caregivers experiencing a severe caregiving burden. Previous studies have consistently found that caregiving burden is prevalent among family caregivers of patients with schizophrenia (Jagannathan et al., 2014; Stanley et al., 2017; Yükü & Derleme, 2017). Comparing caregiving burden in family caregivers of patients with schizophrenia with those who provided caregiving to patients with a general medical condition in India, Stanley et al. (2017) found that the caregiving burden was significantly higher in caregivers of patients with schizophrenia.

Compared to previous studies, caregivers in our study experienced an alarmingly higher burden concerning their caregiving role. According to the results of a systematic review, the caregiver burden in chronic mental diseases, mainly schizophrenia, is at a moderate‐to‐severe level (Yükü & Derleme, 2017). Similarly, a study on a predominantly African ancestry population reported caregiving burden in schizophrenia at mild‐to‐moderate levels (Alexander et al., 2016). In a study in Pakistan, only 15.83% of caregivers of patients with serious mental illness experienced a severe caregiving burden (Siddiqui & Khalid, 2019), while this proportion was 38.27% in our study. This high caregiving burden among caregivers of patients with schizophrenia calls for attention and action by mental healthcare services in Iran. This finding may be partially explained by the long duration of patient disease in our study, indicating more prolonged involvement of the caregivers in caregiving responsibilities. The frequency of caregiving was also high, with 48.8% of participants providing caregiving continuously and daily. Long‐term caregiving can diminish the family's energy and causes despair, helplessness, depression, erosion and the onset or exacerbation of mental disorders in other family members, especially in the patient's parents or spouse, who often assume primary caregiving responsibilities (Iseselo et al., 2016). Family caregivers of patients with mental illness are prone to various emotional, psychological, social and cognitive dysfunctions, which increase their risk of mental health issues (Udoh et al., 2021).

In the regression analysis, the caregiver's age, gender, educational level, job loss due to caregiving, perceived income, relationship with the patient, frequency of caregiving and duration of patient disease remained statistically significant correlates of the caregiving burden. The results are mainly consistent with previous research on the associates of caregivers’ burden in schizophrenia; however, some inconsistent results were observed. We found a statistically significant positive association between the caregiver's age and the caregiving burden, which remained significant in the regression analysis. While this finding is in line with the study by Hidru et al. (2016), other similar studies have reported a non‐significant association between caregivers’ age and the caregiving burden (Arun et al., 2018; Peng et al., 2019; Siddiqui & Khalid, 2019; Stanley et al., 2017; Yükü & Derleme, 2017).

The caregiving burden was higher in female than male caregivers. This finding is consistent with the results of the systematic review conducted by Yükü and Derleme (2017); however, in some individual studies (Arun et al., 2018; Jagannathan et al., 2014; Siddiqui & Khalid, 2019; Stanley et al., 2017), the association between gender and caregiving burden was not statistically significant. Women are usually predominant caregiver for family members with chronic medical conditions, so they could experience high caregiving burden than men (Swinkels et al., 2019).

There was a statistically significant negative relationship between caregivers' education level and perceived caregiving burden. This finding is consistent with previous research showing that caregivers of patients with schizophrenia who have higher education experience less caregiving burden than those with low or no education (Hidru et al., 2016; Jagannathan et al., 2014; Siddiqui & Khalid, 2019; Yükü & Derleme, 2017). Educated caregivers may locate and access information and support resources more efficiently, helping them develop effective strategies to cope with their caregiving stresses (Farzi et al., 2019). Many participants reported inadequate income, which is consistent with previous research. Financial difficulties are related to the extent of caregiving required for patients with schizophrenia. The families experience a crisis in the family system while striving to balance home and work responsibilities and to be able to pay the substantial medical bills (Rahmani et al., 2018). Financial strain is prevalent among these families due to a lack of support from the government (Chen et al., 2019; Tamizi et al., 2020; Weinmann & Koesters, 2016). Overall, our results suggest that caregivers from lower socioeconomic status experienced a more statistically significant caregiving burden. Previous studies, including a systematic review, have also reported an adverse association between socioeconomic status and the caregiving burden (Arun et al., 2018; Hidru et al., 2016; Siddiqui & Khalid, 2019; Yazici et al., 2016; Yükü & Derleme, 2017).

The regression analysis results also showed that the caregiver's relationship with the patient was a statistically significant factor affecting the caregiving burden, with spouse caregivers experiencing a statistically significantly higher burden than parent, child or sibling caregivers. This finding aligns with two previous studies (Stanley et al., 2017; Walke et al., 2018); however, Jagannathan et al. (2014) did not find a statistically significant relationship between these variables. It is argued that spouse caregivers suffer from high emotional burden over time due to their compromised marital relationship (Franza, 2019; Walke et al., 2018). Further, they have to fulfil their household tasks and manage matters brought about by the demands of caregiving by themselves (Schulz et al., 2012), and the lack of proper support adds to their burden (Rahmani et al., 2018; Sharif et al., 2012). However, some spouse caregivers may feel reluctant to ask for help or may not have a choice other than enduring the condition (Ornstein et al., 2019). In societies like Iran, with a predominantly collectivist rather individualistic culture, divorce or separation based on the ill health of spouse is out of bounds (Muoghalu & Jegede, 2010). In such cultures, resolving problems in the family and maintaining family cohesion are highly encouraged (Javidan & Dastmalchian, 2003).

The regression model results also indicated that the caregiving burden was higher in caregivers who provided caregiving constantly and for a long duration. This finding supports previous research that the length of caregiving is inversely related to caregiving burden (Alexander et al., 2016; Hidru et al., 2016; Jagannathan et al., 2014), yet Stanley et al. (2017) did not find a statistically significant association between these variables (Stanley et al., 2017). It is possible that with the increase in the duration of disease and treatment, the disease stabilizes, and caregivers learn to cope with their role (Kate et al., 2013); however, this relationship is not linear, and the caregiving burden intensifies with long‐term and constant caregiving. Patients with schizophrenia require long‐term support from their caregivers, and the burden develops when the demand for caregiving increases; this is particularly true in patients with statistically significant impairments in their functional abilities (Bekdemir & Ilhan, 2019; Pérez‐Cruz et al., 2019).

It is, therefore, important that the caregivers of patients with schizophrenia are supported formally and informally by the family members, relatives, friends and the government to help them maintain their health and well‐being. Experience of loneliness and helplessness by the caregivers can reduce their capacity for providing quality care to the patient, limit their life goals and activities, and lead to mental health issues, such as anxiety and depression (Bowman et al., 2017; Pristavec, 2019). Attention to the needs of caregivers of patients with severe mental diseases, such as schizophrenia, should be a priority for the mental healthcare system in order to maintain the continuity of care for these patients. They should be supported by community services, such as respite care to allow them a break from caregiving responsibilities. Further, having access to free or subsidized mental health care can help alleviate caregiving stress and prevent caregiver burden (Rahmani et al., 2018). The caregivers should be made aware of the caregiving burden and the associated risks and are encouraged to engage in self‐care practice. Educational sessions and participation in peer groups may help the caregivers to share their emotions and experiences and learn from peer experiences. A range of different support services should be available to the caregivers to help them develop effective strategies and build resilience to cope with their ongoing caregiving challenges, maintain a balance between caregiving responsibilities and personal life and empower them to provide quality care to their patients (Hidru et al., 2016; Lök & Bademli, 2021).

### 5.1 Limitation of the study

The study failed to assess a comprehensive list of potential associates of caregiving burden in schizophrenia. Family support, disease severity or the patient's functional disability may also affect the caregiving burden in schizophrenia and need to be examined in future studies. It is also noted that the design of this study prevents establishing cause and effect relationships between the study variables. Finally, there is a need for future research to develop and test effective strategies that can be implemented in mental health services to help reduce the caregiver burden in schizophrenia.

## 6 CONCLUSION

Family caregivers of patients with schizophrenia experienced a high level of caregiving burden. The caregiver's age, gender, education level, job loss due to caregiving responsibilities, income, relationship with the patient and disease duration and frequency of caregiving were identified as statistically significant associates of the caregiving burden in schizophrenia. The results suggest that family caregivers of patients with schizophrenia, particularly those from lower socioeconomic status, should be informed about the risk of caregiving burden and are supported via a range of formal and informal services to develop strategies and build resilience to cope with caregiving challenges.

## 7 RELEVANCE TO CLINICAL PRACTICE

Family caregivers of patients with schizophrenia experience elevated levels of caregiving burden associated with multiple patient and caregiver‐related factors. Interventions to reduce the caregiving burden may include financial support (Tamizi et al., 2020), respite care and access to affordable mental healthcare services (Lök & Bademli, 2021). Family caregivers should be involved in care planning for the patient to enhance their knowledge of the patient's disease states (Hamann & Heres, 2019) and be prepared to take responsibility for caring for a patient with schizophrenia through education and participation in peer groups (Akbari et al., 2018). Caregivers of patients with severe mental diseases, such as schizophrenia, should be supported psychologically, socially and financially to help them cope with their very challenging caregiving role while maintaining their health and well‐being.

## CONFLICT OF INTEREST

The author(s) declared no potential conflicts of interest with respect to the research, authorship and/or publication of this article.

## AUTHOR CONTRIBUTIONS

The first author prepared the proposal for the research project. She also wrote the introduction, method, discussion, conclusion and recommendations of the paper with support from the other authors. The second, third and fourth authors were responsible for the collection of the data for the project. Moreover, the fourth author assisted in the analysis of the data and writing of the results section of the paper. All authors have agreed on the final version and meet at least one of the following criteria [recommended by the ICMJE (http://www.icmje.org/recommendations/)]:

- substantial contributions to conception and design, acquisition of data or analysis and interpretation of data;
- drafting the article or revising it critically for important intellectual content.

## Data Availability

The data of this study cannot be publicly shared due to ethical consideration and protection of the participants' confidentiality.
